# Supplementary material for: Immunogenicity and Immune Memory after a Pneumococcal Polysaccharide Vaccine Booster in a High-Risk Population Primed with 10-Valent or 13-Valent Pneumococcal Conjugate Vaccine: A Randomized Controlled Trial in Papua New Guinean Children
Source: Vaccines (Basel). 2019 Feb 4;7(1):17. doi: 10.3390/vaccines7010017 (PMC6466212; doi:10.3390/vaccines7010017)
Supplement: Supplementary file 1 [file vaccines-07-00017-s001.zip › Supplement materials/Vaccines_Van den Biggelaar_SUPPLEMENT TABLES.docx]

**Table S1. Seroprotection rates at 10 months of age in PPV-vaccinated and PPV-naive children according to priming history with PCV10 or PCV13**

|  | | | **Proportion IgG ≥ 0.35µg/mL** | | | | **Proportion IgG ≥ 1.0 µg/mL** | | | |
| --- | --- | --- | --- | --- | --- | --- | --- | --- | --- | --- |
|  | | | **PPV-naive**  % (95% CI) | | **PPV-vaccinated**  % (95% CI) | | **PPV-naive**  % (95% CI) | | **PPV-vaccinated**  % (95% CI) | |
| **PCV10/PCV13 serotypes** | | | | |  | |  | |  | |
| **1** | **PCV10** | 67% (54-80) | | 100% (100-100) | | 16% (6-26) | | 94% (87-100) | |  |
|  | **PCV13** | 89% (80-98) | | 100% (100-100) | | 31% (18-45) | | 96% (90-100) | |  |
| **4** | **PCV10** | 59% (45-72) | | 94% (87-100) | | 10% (2-18) | | 67% (54-80) | |  |
|  | **PCV13** | 44% (30-59) | | 98% (94-100) | | 13% (3-23) | | 80% (68-91) | |  |
| **5** | **PCV10** | 78% (67-90) | | 98% (94-100) | | 31% (19-44) | | 92% (84-100) | |  |
|  | **PCV13** | 82% (71-93) | | 100% (100-100) | | 40% (26-54) | | 96% (90-100) | |  |
| **6B** | **PCV10** | 82% (72-93) | | 98% (94-100) | | 51% (37-65) | | 88% (79-97) | |  |
|  | **PCV13** | 87% (77-97) | | 100% (100-100) | | 44% (30-59) | | 88% (79-97) | |  |
| **7F** | **PCV10** | 88% (79-97) | | 98% (94-100) | | 35% (22-48) | | 92% (84-100) | |  |
|  | **PCV13** | 96% (90-100) | | 100% (100-100) | | 53% (39-68) | | 90% (81-98) | |  |
| **9V** | **PCV10** | 71% (58-83) | | 100% (100-100) | | 18% (7-28) | | 88% (79-97) | |  |
|  | **PCV13** | 69% (55-82) | | 100% (100-100) | | 20% (8-32) | | 92% (84-100) | |  |
| **14** | **PCV10** | 96% (91-100) | | 100% (100-100) | | 88% (79-97) | | 94% (87-100) | |  |
|  | **PCV13** | 98% (93-100) | | 98% (94-100) | | 82% (71-93) | | 96% (90-100) | |  |
| **18C** | **PCV10** | 88% (79-97) | | 100% (100-100) | | 39% (26-53) | | 89% (81-98) | |  |
|  | **PCV13** | 67% (53-80) | | 98% (94-100) | | 24% (12-37) | | 86% (76-96) | |  |
| **19F** | **PCV10** | 71% (58-84) | | 98% (94-100) | | 84% (74-94) | | 96% (90-100) | |  |
|  | **PCV13** | 96% (90-100) | | 100% (100-100) | | 98% (94-100) | | 98% (94-100) | |  |
| **23F** | **PCV10** | 45% (31-59) | | 94% (87-100) | | 12% (3-21) | | 69% (56-82) ^b^ | |  |
|  | **PCV13** | 51% (37-66) | | 92% (84-100) | | 24% (12-37) | | 69% (56-82) ^b^ | |  |
| **PCV13 serotypes** | | | | |  | |  | |  | |
| **3** | **PCV10** | 41% (28-55) | | 84% (73-94) | | 10% (2-18) | | 55% (41-69) | |  |
|  | **PCV13** | 40% (26-54) | | 86% (76-96) | | 7% (1-14) | | 55% (41-69) | |  |
| **6A^** | **PCV10** | 33% (20-45) | | 57% (43-71) | | 10% (2-18) | | 14% (4-24) | |  |
|  | **PCV13** | 80% (68-92) | | 78% (66-89) | | 29% (16-42) | | 31% (18-44) | |  |
| **19A** | **PCV10** | 78% (67-90) | | 92% (84-100) | | 31% (19-44) | | 57% (43-71) | |  |
|  | **PCV13** | 89% (80-98) | | 100% (100-100) | | 49% (34-63) | | 92% (84-100) | |  |
| **Non-PCV serotype** | | | | |  | |  | |  | |
| **2** | **PCV10** | | 37% (24-51) | | 94% (87-100) | | 37% (24-51) | | 94% (87-100) | |
|  | **PCV13** | | 44% (30-59) | | 94% (87-100) | | 13% (3-23) | | 74% (61-86) | |

The table shows the proportion of children with seroprotective IgG **≥** 0.35µg/mL or ≥ 1.0 µg/mL at 10 months of age for children primed with PCV10 or PCV13 at 1, 2 and 3 months of age, and who did or did not receive a booster dose of PPV at 9 months of age. ^ Serotype 6A is not included in PPV.

**Table S2. Seroprotection rates at 23 months of age in PPV-vaccinated compared to PPV-naive children according to priming history with PCV10 or PCV13**

|  | | | | **Proportion IgG ≥ 0.35µg/mL** | | | | | | **Proportion IgG ≥ 1.0 µg/mL** | | | | |
| --- | --- | --- | --- | --- | --- | --- | --- | --- | --- | --- | --- | --- | --- | --- |
|  | | | | **PPV-naive**  % (95% CI) | | | **PPV-vaccinated**  % (95% CI) | | | **PPV-naive**  % (95% CI) | | | **PPV-vaccinated**  % (95% CI) |  |
| **PCV10/PCV13 serotypes** | | | | |  | | |  | | |  | | |  |
| **1** | **PCV10** | | 67% (54-81) | | | 93% (85-100) | | | 30% (17-44) | | | 52% (37-67) | |  |
|  | **PCV13** | | 78% (65-92) | | | 84% (73-95) | | | 27% (13-41) | | | 41% (26-55) | |  |
| **4** | **PCV10** | | 39% (25-53) | | | 69% (55-83) | | | 9% (1-17) | | | 12% (2-22) | |  |
|  | **PCV13** | | 54% (38-70) | | | 48% (33-62) | | | 24% (11-38) | | | 7% (1-14) | |  |
| **5** | **PCV10** | | 80% (69-92) | | | 90% (82-99) | | | 33% (19-46) | | | 55% (40-70) | |  |
|  | **PCV13** | | 86% (75-98) | | | 93% (86-100) | | | 41% (25-56) | | | 46% (31-60) | |  |
| **6B** | **PCV10** | | 93% (86-100) | | | 93% (85-100) | | | 41% (27-56) | | | 67% (52-81) | |  |
|  | **PCV13** | | 97% (92-100) | | | 93% (86-100) | | | 62% (47-78) | | | 45% (31-60) | |  |
| **7F** | **PCV10** | | 80% (69-92) | | | 95% (89-100) | | | 43% (29-58) | | | 50% (35-65) | |  |
|  | **PCV13** | | 89% (79-99) | | | 91% (82-99) | | | 54% (38-70) | | | 36% (22-51) | |  |
| **9V** | **PCV10** | | 63% (49-77) | | | 88% (78-98) | | | 22% (10-34) | | | 45% (30-60) | |  |
|  | **PCV13** | | 70% (56-85) | | | 82% (70-93) | | | 19% (6-32) | | | 27% (14-40) | |  |
| **14** | **PCV10** | | 100% (100-100) | | | 100% (100-100) | | | 89% (80-98) | | | 95% (89-100) | |  |
|  | **PCV13** | | 100% (100-100) | | | 98% (93-100) | | | 89% (79-99) | | | 86% (76-97) | |  |
| **18C** | **PCV10** | | 61% (47-75) | | | 88% (78-98) | | | 15% (5-26) | | | 21% (9-34) | |  |
|  | **PCV13** | | 70% (56-85) | | | 86% (76-97) | | | 22% (8-35) | | | 25% (12-38) | |  |
| **19F** | **PCV10** | | 98% (94-100) | | | 100% (100-100) | | | 63% (49-77) | | | 88% (78-98) | |  |
|  | **PCV13** | | 97% (92-100) | | | 98% (93-100) | | | 89% (79-99) | | | 86% (76-97) | |  |
| **23F** | **PCV10** | | 52% (38-67) | | | 83% (72-95) | | | 11% (2-20) | | | 12% (2-22) | |  |
|  | **PCV13** | | 65% (49-80) | | | 59% (45-74) | | | 22% (8-35) | | | 18% (7-30) | |  |
| **PCV13 serotypes** | | | | |  | | |  | | |  | | |  |
| **3** | **PCV10** | | 35% (21-49) | | | 33% (19-48) | | | 2% (1-6) | | | 2% (1-7) | |  |
|  | **PCV13** | | 30% (15-44) | | | 27% (14-40) | | | 5% (1-13) | | | 2% (1-7) | |  |
| **6A^** | **PCV10** | | 57% (42-71) | | | 60% (45-74) | | | 15% (5-26) | | | 7% (1-15) | |  |
|  | **PCV13** | | 68% (52-83) | | | 55% (40-69) | | | 30% (15-44) | | | 14% (4-24) | |  |
| **19A** | **PCV10** | | 91% (83-99) | | | 98% (93-100) | | | 59% (44-73) | | | 67% (52-81) | |  |
|  | **PCV13** | | 100% (100-100) | | | 98% (93-100) | | | 70% (56-85) | | | 66% (52-80) | |  |
| **Non-PCV serotype** | | | | |  | | |  | | |  | | |  |
| **2** | **PCV10** | 80% (69-92) | | | 90% (82-99) | | | 37% (23-51) | | | 55% (40-70) | | |  |
|  | **PCV13** | 84% (72-96) | | | 86% (76-97) | | | 27% (13-41) | | | 52% (38-67) | | |  |

The table shows the proportion of children with IgG **≥** 0.35µg/mL or ≥ 1.0 µg/mL at 23 months of age for children primed with PCV10 or PCV13 at 1, 2 and 3 months of age and who did or did not receive a booster dose of PPV at 9 months of age. ^ Serotype 6A is not included in PPV.

**Table S3. Seroprotection rates at 24 months of age, one-month after PPV micro-dose challenge in PPV-vaccinated compared to PPV-naive children according to priming history with PCV10 or PCV13**

|  | | **Proportion IgG ≥ 0.35µg/mL** | | **Proportion IgG ≥ 1.0 µg/mL** | |
| --- | --- | --- | --- | --- | --- |
|  | | **PPV-naive**  % (95% CI) | **PPV-vaccinated**  % (95% CI) | **PPV-naive**  % (95% CI) | **PPV-vaccinated**  % (95% CI) |
| **PCV10/PCV13 serotypes** | | |  |  |  |
| **1** | **PCV10** | 98% (93-100) | 95% (89-100) | 82% (70-93) | 81% (69-93) |
|  | **PCV13** | 97% (92-100) | 95% (89-100) | 86% (75-98) | 67% (52-81) |
| **4** | **PCV10** | 77% (65-90) | 81% (69-93) | 55% (40-69) | 48% (33-63) |
|  | **PCV13** | 92% (83-100) | 83% (72-95) | 62% (47-78) | 29% (15-42) |
| **5** | **PCV10** | 98% (93-100) | 98% (93-100) | 66% (52-80) | 76% (63-89) |
|  | **PCV13** | 97% (92-100) | 98% (93-100) | 70% (56-85) | 50% (35-65) |
| **6B** | **PCV10** | 98% (93-100) | 98% (93-100) | 84% (73-95) | 88% (78-98) |
|  | **PCV13** | 97% (92-100) | 95% (89-100) | 89% (79-99) | 67% (52-81) |
| **7F** | **PCV10** | 100% (100-100) | 98% (93-100) | 82% (70-93) | 69% (55-83) |
|  | **PCV13** | 97% (92-100) | 98% (93-100) | 81% (68-94) | 55% (40-70) |
| **9V** | **PCV10** | 95% (89-100) | 95% (89-100) | 73% (60-86) | 64% (50-79) |
|  | **PCV13** | 95% (87-100) | 95% (89-100) | 59% (44-75) | 50% (35-65) |
| **14** | **PCV10** | 100% (100-100) | 100% (100-100) | 95% (89-100) | 95% (89-100) |
|  | **PCV13** | 100% (100-100) | 100% (100-100) | 97% (92-100) | 98% (93-100) |
| **18C** | **PCV10** | 93% (86-100) | 95% (89-100) | 64% (49-78) | 60% (45-74) |
|  | **PCV13** | 86% (75-98) | 93% (85-100) | 65% (49-80) | 50% (35-65) |
| **19F** | **PCV10** | 100% (100-100) | 100% (100-100) | 89% (79-98) | 98% (93-100) |
|  | **PCV13** | 100% (100-100) | 100% (100-100) | 95% (87-100) | 98% (93-100) |
| **23F** | **PCV10** | 82% (70-93) | 93% (85-100) | 50% (35-65) | 38% (23-53) |
|  | **PCV13** | 84% (72-96) | 71% (58-85) | 65% (49-80) | 29% (15-42) |
| **PCV13 serotypes** | | |  |  |  |
| **3** | **PCV10** | 61% (47-76) | 52% (37-67) | 23% (10-35) | 19% (7-31) |
|  | **PCV13** | 57% (41-73) | 57% (42-72) | 22% (8-35) | 10% (1-18) |
| **6A^** | **PCV10** | 70% (57-84) | 79% (66-91) | 34% (20-48) | 24% (11-37) |
|  | **PCV13** | 76% (62-90) | 69% (55-83) | 49% (33-65) | 17% (5-28) |
| **19A** | **PCV10** | 100% (100-100) | 95% (89-100) | 89% (79-99) | 71% (58-85) |
|  | **PCV13** | 100% (100-100) | 100% (100-100) | 75% (62-88) | 81% (69-93) |
| **Non-PCV serotype** | | |  |  |  |
| **2** | **PCV10** | 98% (93-100) | 93% (85-100) | 82% (70-93) | 76% (63-89) |
|  | **PCV13** | 97% (92-100) | 93% (85-100) | 78% (65-92) | 71% (58-85) |

The table shows the proportion of children with IgG **≥** 0.35µg/mL or ≥ 1.0 µg/mL at 23 months of age for children primed with PCV10 or PCV13 at 1, 2 and 3 months of age and who did or did not receive a booster dose of PPV at 9 months of age. ^ Serotype 6A is not included in PPV.
