# Supplementary figures and images for: Immunogenicity and Immune Memory after a Pneumococcal Polysaccharide Vaccine Booster in a High-Risk Population Primed with 10-Valent or 13-Valent Pneumococcal Conjugate Vaccine: A Randomized Controlled Trial in Papua New Guinean Children
Source: Vaccines (Basel). 2019 Feb 4;7(1):17. doi: 10.3390/vaccines7010017 (PMC6466212; doi:10.3390/vaccines7010017)

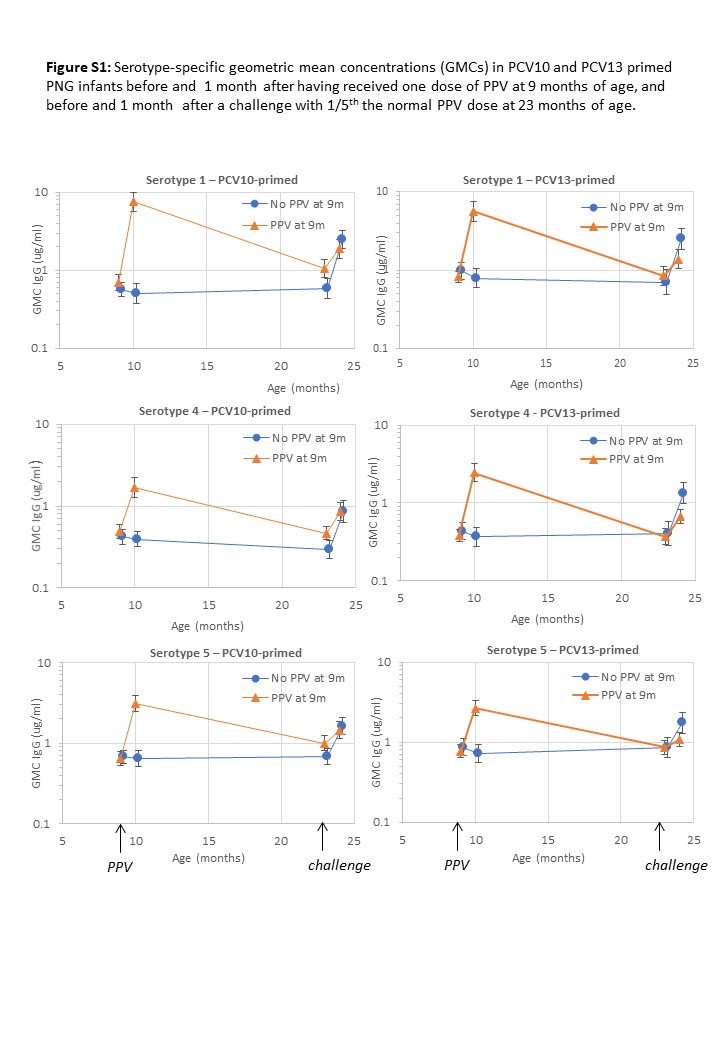

Supplement: Supplementary file 1 [file vaccines-07-00017-s001.zip › Supplement materials/Vaccines_van den Biggelaar_Figure S1/Figure S1 partA.JPG]

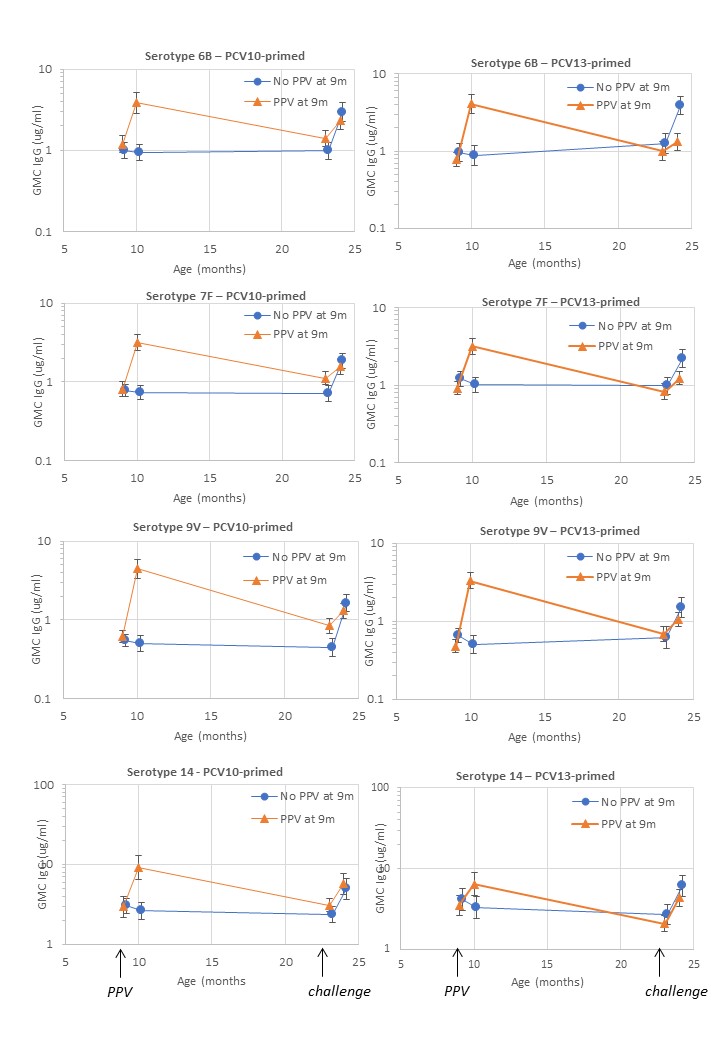

Supplement: Supplementary file 1 [file vaccines-07-00017-s001.zip › Supplement materials/Vaccines_van den Biggelaar_Figure S1/Figure S1 partB.JPG]

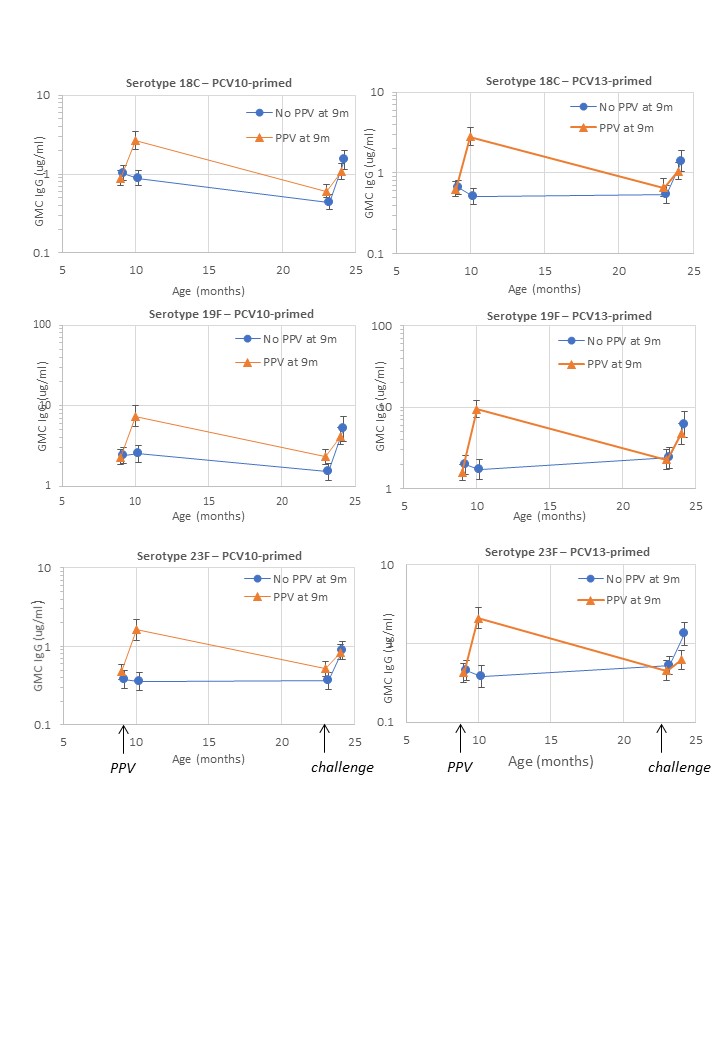

Supplement: Supplementary file 1 [file vaccines-07-00017-s001.zip › Supplement materials/Vaccines_van den Biggelaar_Figure S1/Figure S1 partC.JPG]

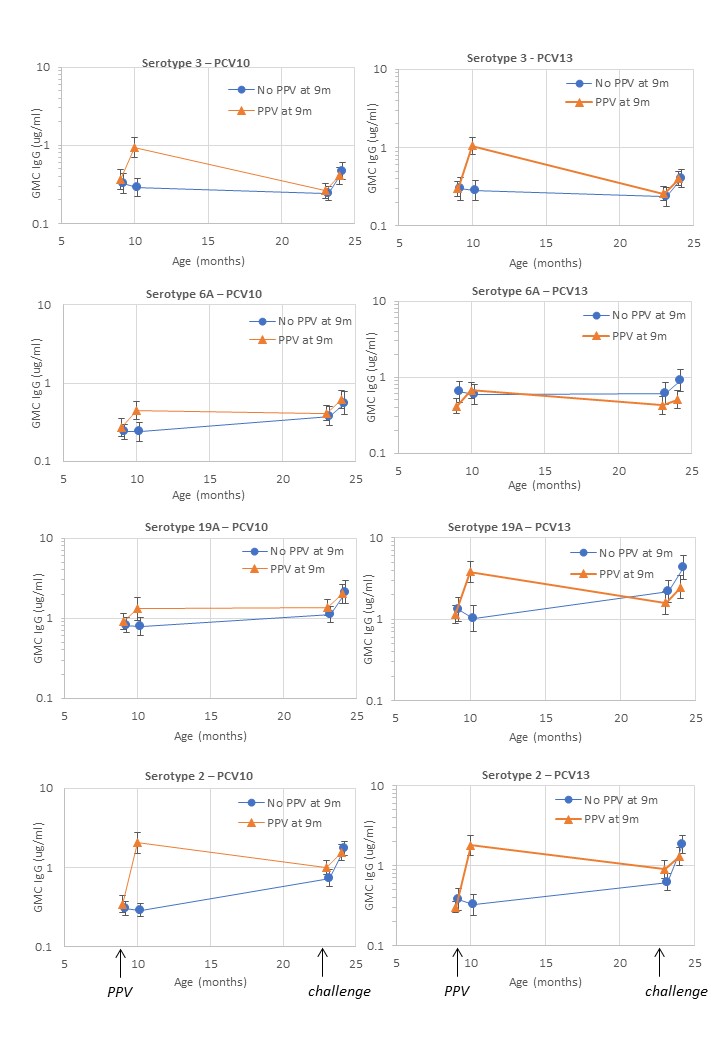

Supplement: Supplementary file 1 [file vaccines-07-00017-s001.zip › Supplement materials/Vaccines_van den Biggelaar_Figure S1/Figure S1 partD.JPG]
